# Expression of *MS4A* and *TMEM176* Genes in Human B Lymphocytes

**DOI:** 10.3389/fimmu.2013.00195

**Published:** 2013-07-15

**Authors:** Jonathan Zuccolo, Lili Deng, Tammy L. Unruh, Ratna Sanyal, Jeremy A. Bau, Jan Storek, Douglas J. Demetrick, Joanne M. Luider, Iwona A. Auer-Grzesiak, Adnan Mansoor, Julie P. Deans

**Affiliations:** ^1^Department of Biochemistry and Molecular Biology, Snyder Institute for Chronic Diseases, University of Calgary, Calgary, AB, Canada; ^2^Department of Medicine, Snyder Institute for Chronic Diseases, University of Calgary, Calgary, AB, Canada; ^3^Department of Oncology, University of Calgary, Calgary, AB, Canada; ^4^Department of Pathology and Laboratory Medicine, University of Calgary, Calgary, AB, Canada; ^5^Calgary Laboratory Services, Foothills Medical Centre, Calgary, AB, Canada

**Keywords:** MS4A, TMEM176, CD20, B lymphocytes, CLL

## Abstract

The *MS4A* gene family in humans includes *CD20* and at least 15 other genes. CD20 exists as homo-oligomers in the plasma membrane, however different MS4A proteins expressed in the same cell may hetero-oligomerize. Given the importance of CD20 in B-cell function and as a therapeutic target, we sought to explore the potential for CD20 hetero-oligomerization with other MS4A proteins. We investigated expression in primary human B-cells of the four *MS4A* genes previously shown to be expressed in human B-cell lines (*MS4A4A*, *MS4A6A*, *MS4A7*, *MS4A8B*), as well as two genes comprising the closely related *TMEM176* gene family, with a view to identifying candidates for future investigation at the protein level. *TMEM176A* and *TMEM176B* transcripts were either not detected, or were detected at relatively low levels in a minority of donor B-cell samples. *MS4A4A* and *MS4A8B* transcripts were not detected in any normal B-cell sample. *MS4A6A* and *MS4A7* transcripts were detected at low levels in most samples, however the corresponding proteins were not at the plasma membrane when expressed as GFP conjugates in BJAB cells. We also examined expression of these genes in chronic lymphocytic leukemia (CLL), and found that it was similar to normal B-cells with two exceptions. First, whereas *MS4A4A* expression was undetected in normal B-cells, it was expressed in 1/14 CLL samples. Second, compared to expression levels in normal B-cells, *MS4A6A* transcripts were elevated in 4/14 CLL samples. In summary, none of the *MS4A/TMEM176* genes tested was expressed at high levels in normal or in most CLL B-cells. *MS4A6A* and *MS4A7* were expressed at low levels in most B-cell samples, however the corresponding proteins may not be positioned at the plasma membrane. Altogether, these data suggest that CD20 normally does not form hetero-oligomers with other MS4A proteins and that there are unlikely to be other MS4A proteins in CLL that might provide useful alternate therapeutic targets.

## Introduction

MS4A is a family of tetraspanning membrane proteins, most of which remain uncharacterized (1, 2). Phylogenetic analysis indicated that the genes encoding these proteins first appeared and expanded in early vertebrates, with rapid diversification such that homologs of some of the human MS4A genes are not found in the mouse and vice versa (3). The founding member of this family, MS4A1 (CD20), is expressed at high levels only in B-lymphocytes, is associated with the B-cell antigen receptor (BCR) (4), functions in BCR-activated calcium influx (5), and is required for optimal humoral immunity (6). Expression of CD20 first occurs during the pre-B stage of development and is lost upon terminal differentiation to plasma cells. CD20 is also expressed at high levels in B-cell lymphomas, and is an effective target for therapeutic depletion of pathogenic B-cells in various disease settings (7–9). Rituximab and other CD20-directed antibody therapeutics bind to epitopes on one or both of the two small extracellular loops between the transmembrane spanning regions (10–13). The short length of these loops brings bound antibodies close to the plasma membrane, a property that is likely to contribute to the efficacy of CD20 as a target (9). Antibody-mediated stabilization of CD20 in lipid rafts (14, 15) appears to allow enhanced complement activation (16), and the slow rate of internalization of antibody-bound CD20 allows prolonged display of Fc regions to Fc receptor-bearing effector cells (17–19). Some or all of these factors are likely to be shared by other MS4A proteins, which could thus be a source of potentially novel immunotherapeutic targets.

CD20 exists as homo-oligomers in the plasma membrane (4, 20), however it has been suggested that different MS4A proteins expressed in the same cell may hetero-oligomerize with one another. Indeed, hetero-oligomerization of murine MS4a4B and MS4a6B has been reported (21), and unusual *MS4A* genes with fused repeating *MS4A* sequences occur in some species (3), suggesting that hetero-oligomerization may be a general feature of MS4A proteins. Given the importance of human CD20 to BCR signaling, humoral immunity, and immunotherapeutic depletion of B-cells, and the potential for other MS4A proteins to hetero-oligomerize with CD20, we sought to identify non-CD20 *MS4A* genes expressed in primary human B-cells. Most of the known *MS4A* genes have expression that is restricted to other tissues, particularly myeloid-lineage cells, testes, lung, and intestinal tissues; however, *MS4A4A*, *MS4A6A*, *MS4A7*, and *MS4A8B* were reported to be expressed in human B-cell lines (2). We investigated the quantitative expression of these 4 *MS4A* genes, as well as two genes comprising the closely related *TMEM176* gene family (3), in human blood and tonsil B-cells with a view to identifying candidates for future investigation at the protein level. A secondary goal was to identify MS4A proteins that might provide alternate therapeutic targets for chronic lymphocytic leukemia (CLL), a B-cell malignancy in which CD20 is expressed at much lower levels than in B-cell lymphomas (22–24). We therefore also investigated expression of *MS4A* and *TMEM176* genes in a group of untreated CLL patients.

## Materials and Methods

### Cell isolation

All samples in this study were obtained by approval of the Conjoint Health Research Ethics Board at the University of Calgary. Blood samples from healthy volunteer donors were obtained from laboratory personnel in the Immunology Research Group, University of Calgary. Tonsils were obtained from patients undergoing tonsillectomy at the Alberta Children’s Hospital. Whole blood from patients with CLL was obtained anonymously as leftover samples from the Flow Cytometry Laboratory of Calgary Laboratory Services at Foothills Medical Center; only samples with dim CD20 expression and ≥96% CD5^+^ B-cells were included in the study. All samples were processed within 24 h of collection. B-cells were purified from whole blood using the RosetteSep™ Human B-cell Enrichment Cocktail (StemCell Technologies, Vancouver, BC, Canada) and from tonsils using a modified RosetteSep™ method (25). CD5^+^ B-cells were prepared from normal blood B-cells using the EasySep Positive Selection kit (StemCell Technologies) with PE-conjugated anti-CD5 (BioLegend, Mississauga, ON, Canada). CD27 positive selection was achieved using CD27-PE with the EasySep PE magnetic selection kit. A minimum of 10^5^ highly purified cells was collected for each population. T cells were purified from whole blood using the Pan T Cell Isolation Kit II (Miltenyi Biotec, Auburn, CA, USA).

### RNA isolation and real-time PCR

Total RNA from blood and tonsil tissue was extracted using Trizol (Invitrogen, Burlington, ON, Canada). RNA from a variety of human tissues was purchased from Clontech, Mountain View, CA. 1 μg of total RNA was converted to cDNA using SuperScript III Reverse Transcriptase (Invitrogen, Burlington, ON, Canada) and oligo dT primers in a volume of 20 μl. The cDNA sample was diluted to 100 and 1 μl of this was used as template for quantitative real-time PCR (qPCR) using SybrGreen (Applied Biosystems, Streetsville, ON, Canada), the primer sets shown in Table 1, and the ABI 7000 machine (Applied Biosystems) in the 96-well format. Reactions were performed in duplicate under the following conditions: 95 °C for 10 min followed by 40 cycles of 60 °C for 1 min and 95 °C for 15 s. Each run was concluded with a melting curve analysis to verify the specificity of the PCR product. Amplification of specific products was further validated during PCR optimization by gel purification and sequencing. Amplification efficiencies, calculated using 10-fold dilutions (10^−1^ to 10^−5^) of cDNA prepared from tonsil B-cell RNA, were close to 2.0 in every case (Table 1). Data were normalized to an endogenous control, *ACTB* (β-actin). mRNA levels in each sample were thus expressed as the difference between threshold values (ΔCT) for the gene transcript of interest and *ACTB*, using the equation Ratio = (E_target_)^ΔCP^ target(control − sample)/(E_ref_)^ΔCP^ ref(control − sample) (26).

**Table 1:** **Human MS4A/TMEM176 qPCR primer sequences and properties**.

Primer name	Accession number	Sequence	Melting temp.	AE^1^
qhMS4A1F	NM_152866.2	CAC CCA TCT GTG TGA CTG TGT G	68	1.98
qhMS4A1R		AGT TTT TCT CCG TTG CTG CC	60	
qhMS4A4AF	NM_148975.2	TGG CTG TCA TAC ATT CAC ATC TG	60	2.04
qhMS4A4AR		CCA TAC ACA TCA TTG TTA TTC CCA	60	
qhMS4A6AF	NM_152852.2	CAC GCA GAA ATC AAA GTT ATT G	60	1.98
qhMS4A6AR		TGG GTA AGC AGA GTT CAA CAG TG	68	
qhMS4A7F	NM_021201.4	CAC CAA AGG GCA TCA CTA TCC	64	1.92
qhMS4A7R		GAA ATC AAC AGG CAA CAC AGG	62	
qhMS4A8BF	NM_031457.1	GAT CTC TCT CCG TGG CAG C	60	2.19
qhMS4A8BR		TGA CGA TGT TCA AGC CCA AAC	60	
qhTMEM176AF	NM_018487.2	GAG TCC AGA AGA AGT CAG AAG GC	70	2.06
qhTMEM176AR		AAG CAG CAG AAT CCA GAC ACC	64	
qhTMEM176BF	NM_014020.3	GGC AGA AGG AGG AGT GTA GAG C	70	1.98
qhTMEM176BR		CAG GAA CAG GGC ACG GAT T	60	
qhB-ACTINR	NM_001101.3	GTG TTG GCG TAC AGG TCT TTG	64	2.01
qhB-ACTINF		CAC TCT TCC AGC CTT CCT TCC	66	

*^1^Amplification efficiency*.

### cDNA constructs and transfections

The human *MS4A4A*, *MS4A6A*, *MS4A7*, and *MS4A8B* coding sequences were cloned and inserted into the multicloning site (MCS) of the pEGFP-C1 vector (Clontech, Mountain View, CA, USA). BJAB cells were electroporated at 340 V and 950 μF (Gene Pulser II, Bio-Rad, Hercules, CA, USA) with 20 μg of cDNA. GFP-positive cell lines were generated in 1 mg/ml Geneticin (Life Technologies). BJAB cells stably expressing GFP-CD20 were previously established (15).

### Immunofluorescence microscopy

Cells were incubated with antibodies either before or after fixation in 4% paraformaldehyde (PFA, Electron Microscopy Sciences, Hatfield, PA, USA) at room temperature for 15 min. Antibodies against EEA1, golgin 95, golgin 97, and giantin were provided by Dr. Marvin Fritzler (University of Calgary, AB, Canada). CD20 mAb 2H7 was provided by Dr. J. Ledbetter (Bristol-Myers Squibb, Seattle, WA, USA). Cy3-conjugated F(ab′)_2_ goat anti-mouse IgG was purchased from Jackson ImmunoResearch Laboratories Inc. (West Grove, PA, USA). Nuclei were stained with 4′-6-Diamidino-2-phenylindole (DAPI, Sigma). Fluorescence imaging was done with a Leica DM RXA microscope (Rockleigh, NJ, USA) attached to a 14-bit cooled CCD camera (Princeton Instruments, Monmouth Junction, NJ, USA). Digital deconvolution was performed using the nearest neighbor algorithm, Microtome for Windows (Vaytek, Fairfield, IA, USA).

## Results

**Figure 1:**
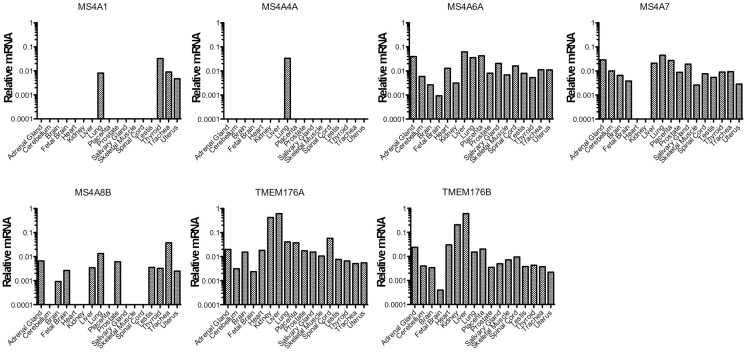
***MS4A*/*TMEM176* mRNA levels in non-hematopoietic human tissues**. cDNA was prepared from tissue RNA samples in the human total RNA master panel II (Clontech). Quantitative PCR was performed to amplify *MS4A1*, *MS4A4A*, *MS4A6A*, *MS4A7*, *MS4A8B*, *TMEM176A*, *TMEM176B*, and *ACTB* cDNA using primer sets with equivalent amplification efficiencies (Table 1). Vertical axes show mRNA levels relative to *ACTB*.

**Figure 2:**
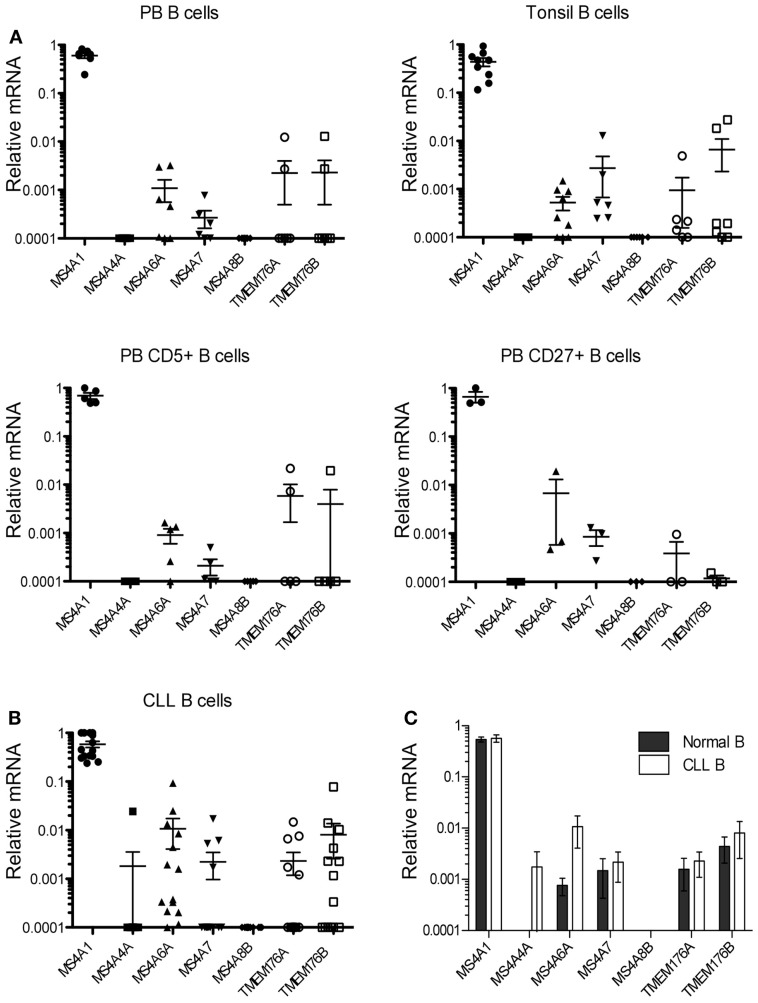
***MS4A/TMEM176* mRNA levels in normal and CLL B-cells**. cDNA was prepared from freshly isolated normal B-cell samples and CLL B-cell samples. Quantitative PCR was performed to amplify *MS4A1*, *MS4A4A*, *MS4A6A*, *MS4A7*, *MS4A8B*, *TMEM176A*, *TMEM176B*, and *ACTB* cDNA as in Figure 1. Vertical axes show mRNA levels relative to *ACTB*. Each data point represents one donor or patient sample. **(A)** peripheral blood (PB) B-cells isolated from seven healthy donors, B-cells isolated from tonsillar tissue from seven patients, CD5+ B-cells sorted from five donor blood samples, CD27+ B-cells sorted from three donor blood samples, and **(B)** 14 CLL B-cell samples. Means and SEM are indicated by the horizontal bars. **(C)** Quantitative PCR data from the 14 blood and tonsil B-cell samples were compared with data from the 14 CLL B-cell samples, with means and SEM shown. Data were analyzed by Mann–Whitney U and there was no statistical significance between the groups (*p* > 0.05).

The *MS4A4A*, *MS4A6A*, *MS4A7*, and *MS4A8B* genes were selected for analysis in this study on the basis of evidence that they are expressed in B-cell lines (2). *MS4A* genes with expression known to be limited to myeloid-lineage cells or non-hematopoietic tissues were excluded. *MS4A1* (CD20), which is expressed at high levels in mature B-cells, was included for comparison. In addition, we examined expression of the *MS4A*-related genes, *TMEM176A* and *TMEM176B*, which have not previously been assessed in human B-cells. The levels of expression were assessed using quantitative RT-PCR (qPCR), with validation of the primer sets done by confirming the expected sizes and sequences of the amplified products. Amplification of each gene was compared to that of *ACTB* (β-actin) and shown on the *y*-axes in Figures 1 and 2 as Relative mRNA.

We first examined the expression of the *MS4A* and *TMEM176* genes in non-hematopoietic tissues (Figure 1). Detection of *MS4A1* (CD20) transcripts in the lung, trachea, thyroid, and uterus is probably indicative of blood contamination or infiltration of these tissues. Among non-hematopoietic tissues, *MS4A4A* detection was limited to the lung. Considering that *MS4A1* (CD20) transcripts were also amplified from the lung, *MS4A4A* may be expressed on a hematopoietic cell type in that tissue. *MS4A8B* was expressed most highly in the lung and trachea, consistent with a previous report (27), and was also expressed in a few other tissues but with a more restricted profile than the remaining genes, *MS4A6A*, *MS4A7*, *TMEM176A*, and *TMEM176B*, which were detected broadly in non-hematopoietic tissues (Figure 1).

We then examined expression of the *MS4A4A* and *TMEM176* genes in B-cells isolated from multiple donor samples of peripheral blood and tonsil tissues. As expected, *MS4A1* (CD20) transcripts were always detected at high levels (Figure 2A). Surprisingly, however, we detected only relatively low levels of transcripts for any of the six new *MS4A*/*TMEM176* genes in either tonsil or peripheral blood B-cells. Indeed, *MS4A4A* and *MS4A8B* transcripts were not detected in any sample (Figure 2A). *MS4A6A* and *MS4A7* transcripts were detected in most B-cell samples but at two to four orders of magnitude lower than *MS4A1* (CD20). *TMEM176A* and *TMEM176B* transcripts were found in a minority of blood and tonsil B-cell samples. The expression of *MS4A* and *TMEM176* genes in CD5+ blood B-cells and CD27+ (memory) blood B-cells was examined with similar findings: no detection of *MS4A4A* or *MS4A8B*, and low or no detection of transcripts from the remaining genes (Figure 2A).

Data were obtained for 14 blood samples obtained from CLL patients (Table 2) and tested similarly for the expression of the *MS4A4A* and *TMEM176* genes (Figure 2B). *MS4A1* (CD20) transcripts were detected at high levels in every sample, as expected (24). *MS4A4A* expression, which was undetected in normal B-cells, was detected in a single sample. Notably, *MS4A6A* transcripts were detected at higher levels in 4/14 of CLL samples than in all but one of the 14 normal blood or tonsil B-cell samples, the single exception being one of the blood CD27+ samples. However, the differences in *MS4A6A* expression between CLL and normal B-cells were not statistically significant (Figure 2C). *MS4A8B* was not detected in any CLL sample. Expression of *MS4A7* and the *TMEM176* genes was either low or undetected in CLL samples, as it was in normal B-cells.

**Table 2:** **Chronic lymphocytic leukemia patients**.

	Age	Gender	Months since diagnosis^1^	WBC count	RAI stage	Prior treatment
01	55	F	1	9.5	1	No
02	62	M	0	8	1	No
03	71	F	0	5	0	No
04	58	F	1	13	0	No
05	69	M	0	5	0	No
06	78	F	0	na^2^	na^2^	No
07	66	M	0	12	2	No
08	47	F	50	24	0	No
09	83	F	0	79	0	No
10	79	M	0	12	1	No
11	49	F	0	10	0	No
12	54	M	0	33	0	No
13	71	M	0	20	0	No
14	58	F	0	na^2^	1	No

*Data obtained from Calgary Laboratory Services with ethical approval*.

*^1^Months between diagnosis and time of testing; ^2^na, not available*.

Statistical analysis confirmed that the levels of all *MS4A* and *TMEM176* transcripts in CLL were not different than levels found in normal B-cells (Figure 2C).

**Figure 3:**
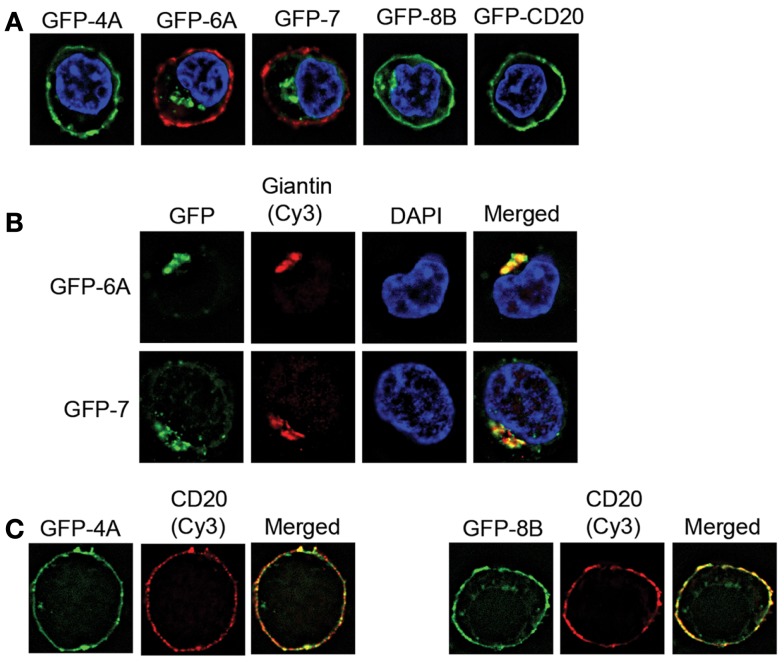
**Intracellular localization of GFP-tagged MS4A6A and MS4A7**. BJAB cell lines were derived that express GFP-tagged MS4A4A, MS4A6A, MS4A7, MS4A8B, and CD20. **(A)** Immunofluorescence images are of individual cells representative of>100 cells observed in at least two independent experiments for each cell line. Cells were fixed and nuclei stained with DAPI. In the case of GFP-MS4A6A and GFP-MS4A7 the cells were counterstained with mouse anti-human CD20 (2H7) and Cy3-conjugated F(ab′)_2_ anti-mouse IgG to delineate the plasma membrane. **(B)** Cells expressing GFP-MS4A6A or GFP-MS4A7 were fixed, permeabilized, and stained with rabbit anti-giantin and Cy3-conjugated anti-rabbit IgG to mark the trans-Golgi. Control experiments with non-specific primary antibody confirmed the specificity of the fluorescent signals obtained. Single-labeled control samples were imaged separately to confirm negligible bleed-through of fluorophores to the other channel. Nuclei were stained with DAPI. Results are representative of>100 cells observed in at least two independent experiments. **(C)** Cells expressing GFP-MS4A4A or GFP-MS4A8B were stained for CD20 as in**(A)**.

The data obtained from normal primary B-cells indicated some expression of *MS4A6A* and *MS4A7* in the majority of the samples tested (Figure 2A). In order to assess the potential for the corresponding MS4A proteins to hetero-oligomerize with CD20, we expressed them as GFP fusion proteins in the human BJAB cell line and examined their subcellular localization by fluorescence microscopy. GFP-tagged MS4A4A, MS4A8B, and CD20 were expressed for comparison. Cells were fixed and nuclei stained with DAPI. GFP-MS4A4A and GFP-MS4A8B, like GFP-CD20, appeared to be expressed at the plasma membrane (Figure 3A) and this was confirmed by visualizing the plasma membrane using anti-CD20 (Figure 3C). In contrast, GFP-MS4A6A and GFP-MS4A7 were localized intracellularly in the perinuclear space (Figure 3A). To characterize the intracellular compartment, we stained early endosomes, cis-Golgi, cis/medial Golgi, and trans-Golgi compartments using antibodies detecting EEA1, golgin 97, golgin 95, and giantin, respectively. Both GFP-MS4A6A and GFP-MS4A7 co-localized with giantin, indicating that they were concentrated in the trans-Golgi complex (Figure 3B); no colocalization with EEA1, golgin 97, or golgin 95 was observed (data not shown).

## Discussion

This report describes the results of our investigation into the expression of *MS4A4A*, *MS4A6A*, *MS4A7*, *MS4A8B*, and both of the *MS4A*-related *TMEM176* genes in normal B-cells and in CLL. Unlike *MS4A1* (CD20), which is consistently and highly expressed in both normal and CLL B-cells at the mRNA level, the other *MS4A*/*TMEM176* genes tested were either not expressed or expressed at relatively low and/or variable levels. *MS4A4A* and *MS4A8B* genes were not expressed in normal blood or tonsillar B-cells; this was a surprising result given a previous report of their expression in B-cell lines (2), however, antibodies generated against the corresponding proteins confirmed that MS4A4A and MS4A8B are not expressed in mature human B-cells (not shown).

*MS4A6A* and *MS4A7* transcripts were detected at low levels, generally 200–400 times lower than *MS4A1* (CD20). A recent global analysis of RNA and protein levels showed that they are significantly correlated in ∼40% of comparisons, higher than previous estimates but clearly not absolute (28). Although the overall correlation is relatively poor, unstable mRNAs tend not to generate stable proteins except in restricted categories of proteins such as those regulating RNA processing (28). Thus, it is unlikely that very low levels of mRNAs would generate high levels of membrane proteins. We could not examine endogenous MS4A6 and MS4A7 as reliable antibodies were not available, however we found that neither of these proteins localized to the plasma membrane when expressed as GFP fusion proteins in a human B-cell line. This is unlikely to be an artifact of GFP fusion as a similarly constructed GFP-CD20 protein was properly localized to the plasma membrane, as were GFP-MS4A4A and GFP-MS4A8B. Known intracellular localization of MS4A3 (HTm4) provides a precedent for intracellular localization of some other MS4A proteins (29). It is possible that MS4A6A and/or MS4A7 are mobilized to the plasma membrane under certain conditions, however, our results suggest that neither MS4A6A or MS4A7 is found in sufficient abundance or in the right subcellular compartment to normally form hetero-oligomers with CD20 or to be considered as an alternate therapeutic target for B-cell depletion.

*TMEM176A* and *TMEM176B* transcripts were undetected or detected at only trace levels in most samples of normal blood or tonsillar B-cells. From this it is clear that neither corresponding protein is normally expressed in all mature human B-cells, although expression could be upregulated under certain conditions. Similar to *MS4A6A* and *MS4A7*, tissue expression was broad for both *TMEM176* genes suggesting generalized rather than cell-type specific functions, and negating their potential utility as therapeutic targets.

The expression profiles of the tested *MS4A* and *TMEM176* genes in CLL were generally similar to that in normal B-cells, however there were a few CLL samples in which expression levels of one of the *MS4A* genes was unusually high. *MS4A4A*, which was never detected in blood or tonsil B-cells, was expressed at a high level in one CLL sample. This sample was from a patient who presented with RAI stage II CLL at the time of diagnosis when the sample was collected (Table 2), however studies on much larger, defined patient populations would need to be performed to determine the significance, if any, of this observation. *MS4A6A* was more highly expressed in about 1/3 of CLL samples than in any of the normal B-cell samples, but this difference did not reach statistical significance (Figure 2C). Our study was not designed to test the potential correlation of differential *MS4A*/*TMEM176* gene expression with clinical indicators of prognosis and no conclusions about the significance of these observations can be made at this time. However, testing the protein expression of MS4A4A and MS4A6A in a large cohort of defined CLL samples would be an important direction when antibodies suitable for flow cytometry become available.

In conclusion, we searched for and did not find evidence for *MS4A4A*, *MS4A6A*, *MS4A7*, *MS4A8B*, or *TMEM176* transcripts likely to generate proteins in sufficient quantity to form hetero-oligomers with the abundant CD20 molecules expressed in all normal mature B-cells. *MS4A4A* and *MS4A8B* were not detected. *MS4A6A* and *MS4A7* transcripts were detected in most normal B samples but at very low levels and the corresponding proteins are probably not normally expressed at the plasma membrane. *TMEM176A* and *TMEM176B* transcripts were only detected in a minority of B-cell samples. Available data for other *MS4A* genes shows expression restricted to myeloid-lineage cells, testis, lung, and intestinal tissues. Therefore, in the absence of evidence of other MS4A proteins co-expressed with CD20 at the plasma membrane, it is most likely that CD20 normally exists in homo-oligomeric form and that alternate therapeutic targets for B-cells are unlikely to be found in the MS4A family. Novel targets are more likely to be found among members of the unrelated but structurally similar tetraspanin superfamily (30). Although broad tissue expression of tetraspanin proteins such as CD9 and CD81 limits their potential in this regard, high expression of CD37 in B-cells makes it a promising target for CLL and other B-cell cancers (31–34).

## Conflict of Interest Statement

The authors declare that the research was conducted in the absence of any commercial or financial relationships that could be construed as a potential conflict of interest.
